# Community service nurses in primary healthcare clinics: Experiences of supervision and support from professional nurses

**DOI:** 10.4102/hsag.v27i0.1738

**Published:** 2022-10-26

**Authors:** Ayanda Zambodla, Margaret Williams, Esmeralda Ricks

**Affiliations:** 1 Department of Nursing, Faculty of Health Sciences, Nelson Mandela University, Port Elizabeth, South Africa; 2 Department of Nursing, Faculty of Community and Health Sciences, University of the Western Cape, Cape Town, South Africa

**Keywords:** primary healthcare, community service, community service nurse, supervision, support

## Abstract

**Background:**

Newly qualified nurses in South Africa are required to undertake a compulsory 1-year community service (CS) in a public healthcare facility. They are not yet competent to work alone and require supervision and support from senior professional nurses.

**Aim:**

This study aimed to explore and describe the experiences of community service nurses (CSNs) regarding supervision and support from professional nurses at primary healthcare clinics (PHC).

**Setting:**

The study was conducted with CSNs who were working in PHC clinics in Nelson Mandela Bay (NMB).

**Methods:**

A qualitative, explorative, descriptive and contextual study was conducted using semi-structured interviews with 10 CSNs. Purposive sampling was used. Data were analysed using Tesch’s coding method of content analysis.

**Results:**

Two themes and six sub-themes emerged, the key themes being participants’ diverse needs related to CS placement at PHC clinics and participants’ social interaction with the professional nurses during CS.

**Conclusion:**

Supervision and support for the CSNs during their CS rotation was inadequate at most clinics in this study. Recommendations, based on findings, were made for an improved CS experience and further research.

**Contribution:**

The CSNs require supervision and support in the CS year, particularly in PHC clinics, clearly presented in this study. The findings of the study can be used to improve the experience of CS for CSNs in the PHC setting in NMB.

## Introduction and background

Community service (CS) for health professionals is a strategy that commenced in 1998 as part of the South African government’s plan to address the problem of inequality in the distribution of healthcare services and health professionals, particularly in rural areas (Reid 2018). The two objectives of CS are to provide for the wider distribution of healthcare professionals in South Africa and to allow healthcare professionals to gain experience (Reid 2018).

Primary healthcare (PHC) is pivotal to the suggested united healthcare system for South Africa, underpinned by the intended National Health Insurance initiative. Therefore, the PHC nurse must be optimally capacitated to provide this service (Republic of South Africa 2018). Community Service Nurses (CSNs) are often deployed in PHC settings; however, they have not yet developed clinical competence, which requires time, supervision and support. Benner (1984) states that the formation of professional values and identity occurs over time with transformation through experiential learning. Hill (2017) defines experiential learning as learning by doing, experiencing, practical learning and reflection. Sand et al. (2014) consider that experiential learning is significant in connecting the knowledge with the classroom and the clinical setting. The expectation is that the novice-nurses have the basic professional skills set on which to build experiential learning.

For nurses, CS is a transition period between the end of their student training to full employment, allowing the development of essential skills before assuming full responsibility as a professional nurse (Govender, Brysiewicz & Bhengu 2015). To develop professionally, supervision and support is required from professional nurses in the clinical settings where CSNs work.

Support for CSNs requires supervision by experienced, qualified colleagues and being trusted to perform relevant tasks (Abiodun et al. 2019; Roziers, Kyriakos & Ramugondo 2014). Clinical supervision is part of the scope of practice of a registered nurse in South Africa, as articulated in Regulation 786 of the Nursing Act, No. 33 of 2005. Under provision 4.4 (j) of Regulation 786, it is stated that the professional nurse should actively engage in the education and training of learners in the healthcare system (South African Nursing Council Nursing Act 2005).

To maximally strengthen the PHC system, it is pertinent to understand the role of the PHC nurses and CS nurses. According to Michael et al. (2018), PHC in South Africa is nurse-led and doctor-supported and the number, competency and effectiveness of nurses are critical in determining the quality of care. Registered nurses in South Africa working in PHC clinics work as independent practitioners, managing minor ailments, chronic illnesses and prescribing treatments as per treatment guidelines.

The role of the PHC nurse requires precise history taking, diagnostic and clinical management skills (Strausser 2015). The CSN being a newly qualified nurse, once placed in the PHC setting, is required to perform these functions. A study by Morolong and Chabeli (2015) revealed that newly qualified nurses often lack the basic knowledge, skills, attitudes and values of the nursing profession as well as critical thinking skills in their approach to providing quality patient care. These are acquired through observation, supervision and support from senior and experienced colleagues.

Govender et al. (2015) reported that newly qualified nurses in KwaZulu-Natal, South Africa struggled to integrate into the health institutions in which they were employed because of an absence of formal structures, including supervision. This resulted in inconsistencies in the roles the CSNs could fulfil and the roles they were assigned in the PHC facilities.

The study was underpinned by Kotzé’s accompaniment theory. In this study, principles of the theory have been applied in the context of CSNs in the PHC setting who require systematic interventions of supervision and support from professional nurses until they are competent and are able to work independently (Kotze 1998).

### Statement of problem

The researcher worked in PHC clinics where it was observed that there was a reluctance on the part of the nursing staff to orientate, support and supervise the CSNs. The primary aim of CS for healthcare professionals is an opportunity to develop skills, acquire knowledge and gain experience (Nkabinde et al. 2013; Nkoane & Mavhandu-Mudzusi 2020).

According to Guise (2012), core norms and standards for clinics developed by the Department of Health state that the safety of the patient is paramount, and no member of staff should undertake tasks unless they are competent to do so. The South African Department of Health (2006) states that newly qualified CSNs should not be working without supervision.

Literature indicated that not all CSNs received supervision and support during the CS year.

### Research question

The following research question directed this study.

How did CSNs who were placed in PHC facilities in Nelson Mandela Bay (NMB) experience the supervision and support provided by professional nurses?

### Purpose

This study aimed to explore and describe the experiences of CSNs, regarding the supervision and support received from professional nurses at PHC facilities in NMB.

### Contribution to the field

The unique contribution of this article is the voices of the participants as they relate their experiences of supervision and support received whilst working their CS year in PHC clinics in NMB. The findings could inform relevant stakeholders about the challenges experienced by participants.

## Research design and methods

### Research setting

The PHC clinics in NMB used in the study were mainly situated in sub-economic areas with diverse populations regarding age distribution, race and gender. Staff were heterogonous in terms of qualifications, age and ethnicity. Nelson Mandela Bay Health District (NMBD) comprises 50 PHC facilities. A total of 20 CSNs were appointed to 16 of these PHC facilities for their CS year in the 2016–2017 CSNs cycle.

### Study approach and design

A qualitative, exploratory, descriptive contextual study was conducted using semi-structured face-to-face interviews to allow for broad-based questions and the emergence of new information unique to the experiences of the participants with regard to their CS year in PHC clinics (Creswell & Creswell 2018; Streubert & Carpenter 2011).

### Population and sampling

The research population comprised CSNs working in various PHC facilities in NMB. A criterion-based purposive sampling strategy was used to select the participants (Kumar 2014). The criterion for inclusion in the study was CSNs who commenced their CS in the 2016–2017 cycle. Ten participants who matched the selection criterion were selected from nine PHC facilities in NMB.

### Data collection methods

Data collection commenced once ethical approval was received. Semi-structured individual interviews were used to collect the research data (Rebar et al. 2014). Participation in the study was voluntary and the interviews were conducted by A.Z. in English and averaged 45 min in duration. The district office managers in NMB acted as gatekeepers to gain access to the CSNs. All interviews were audio-recorded and transcribed verbatim. All interviews were conducted in a private room in the clinic. The central open-ended question presented to the participants was: ‘Can you tell me about your experiences regarding supervision and support received from professional nurses during your CS placement in PHC clinics?’. Data were collected until data saturation occurred when no new information emerged. The researcher took field notes based on her observations of the interviewee and details of the interaction. The field notes supplemented the data collected during the semi-structured interviews.

### Data analysis

Tesch’s open coding method was performed as outlined by Creswell and Creswell (2018), as content analysis was conducted to analyse the data. Transcribed data were read and re-read to make sense of the whole and coded manually. Themes were created from the identified sub-themes, and anecdotal notes were used to assist during the latter process as emphasised in Brink, Van Der Walt and Van Rensburg (2018). An independent coder verified the coding and themes using Atlas. Ti, version 8.4.3 qualitative data analysis software package. A consensus meeting was held between the researchers and independent coder to review themes and make adjustments where necessary. Literature was used to support the research findings (Creswell & Creswell 2018; Green & Thorogood 2018).

### Trustworthiness

In this study, Guba’s model of trustworthiness was applied. Credibility was facilitated by ensuring that the researchers’ findings were compatible with the experiences of the participants (Connelly 2016; Streubert & Carpenter 2011). The credibility of the data was endorsed through the audio-recorded interviews and verbatim transcription. Triangulation was ensured through semi-structured interviews and supplementary descriptive field notes (Noble & Heale 2019; Polit & Beck 2016). Rich, thick descriptions of the setting and participants’ demographics facilitated decisions regarding the transferability of the findings (Creswell & Creswell 2018; Johnson, Adkins & Chauvin 2019; Shezi 2014). An audit of the research process and results facilitated dependability (Streubert & Carpenter 2011). Confirmability was established by using direct quotes from the participants in the research report (Polit & Beck 2016).

### Ethical considerations

Ethical clearance to conduct this study was obtained from the Faculty Post Graduate Studies Committee of Nelson Mandela University (reference number: H18-HEA-NUR-005). Participation was voluntary, and participants were informed of their right to withdraw from the study at any time. All participants provided their written consent prior to the commencement of interviews, which included permission to audio-record. The ethical principles of no harm, confidentiality, privacy and anonymity and justice were upheld; the participants and facilities were not identifiable, and the selection of participants was based on the study inclusion criteria (Streubert & Carpenter 2011).

## Results

The participants age ranged from 22 to 40 years. Seven participants were female, and three were male. The male participants had previous work experience outside the nursing discipline, while the female participants did not. Six participants had a 4-year diploma, and four had a 4-year degree in nursing. Eight participants requested a PHC facility as their first choice for CS. Nine participants completed CS in their hometown.

Two themes and six sub-themes emerged from the data (see Table 1-A1).

### Theme 1: The participants experienced diverse needs related to their community service placement at primary healthcare clinics

Participants, having discussed their experiences with other CSNs, observed that their experience of supervision and support differed and revealed a lack of a uniform structured orientation programme for CS in all PHC clinics. They reported the need for orientation, adequate supervision, information and experiential learning through being rotated to various departments within the PHC facility.

#### Sub-theme 1.1: Participants experienced the need for orientation

Participants expressed that they had expectations of not being orientated to their new work environment before commencing work:

‘So, what I expected was that orientation to what is happening in the facility [*be provided upon arrival*]… [*I*] think immediately after or just before an individual start CS, I think there should be a programme that one could join, you know, to give CSN advice … what is expected of you ….’ (Participant 7:2)

‘I think that first 2 weeks of orientation, before we go to the facilities, we should get all the trainings.’ (Participant 6:4)

One of the participants spoke of the lack of structured orientation in the CS programme:

‘… I found out that there is no formal [*structured*] orientation programme in place, for people that are comserving.’ (Participant 3:3)

Some participants mentioned that they received some form of orientation:

‘Orientation, I meant… okay, …I was orientated … around the facility, yes when I got there….They orientated me with regard to all the guidelines, so I could be familiar with the work that I am going to do.’ (Participant 10:2)

#### Sub-theme 1.2: Participants experienced the need for adequate supervision

Participants indicated that their expectations regarding supervision and support from the professional nurses at the PHC clinics were not met. They expected the professional nurses in the PHC clinics to oversee everything that they did and indicated that CSNs should not be left on their own:

‘But when it comes to supervision, I did not get the supervision that I thought I was going to get during my comserve (CS).’ (Participant 3:9)

‘I can say it’s not what I expected. It is always said that when you’re CS, you have to work under supervision, you bypass [*check with*] a professional nurse with whatever you do: the nurse must be there to witness whatever you do.’ (Participant 7:1)

Participants expressed feeling anxiety over working unsupervised:

‘I didn’t have any support or supervision, it was just chaotic for me. I felt like I was just…I don’t know, I just felt like giving up, it’s like if anything goes wrong here, its mother and its baby, unborn baby and it’s high risk. Nobody’s willing to help me.’ (Participant 2:2)

Participants reported that a combination of poor staffing levels and unrealistic expectations of the skills level of CSNs contributes to the CSN working unsupervised:

‘… but the problem [*of inadequate supervision*] boils down to the shortage of staff, because there…even the facility managers they are doing services instead of administration and supervision in the clinics… I feel that the com serves are being ignored.’ (Participant 3:10)

In some instances, mistakes were made because of having to work alone, without supervision:

‘… [*A*]nd as time went by as I started to get into it and understood the work, I realised that I had made a lot of mistakes, rookie mistakes. I would say, because I did not get the supervision that was necessary.’ (Participant 3:1)

A participant stated that after attending in-service training he was able to reflect on his nursing practice and identify errors he made, which he attributed to being as a result of not having adequate supervision and guidance from the professional nurses:

‘When I went for trainings, I got to see that, no man… it was not really what I was supposed to do. But I was fortunate that nothing happened to the patient.’ (Participant 1:2)

The participants were aware of the potentially negative consequences of working unsupervised but had no options. A participant shares her perception as to why she was not assisted:

‘It could be the case where the very same nurse who is experienced now was …she is treating me the way that she was treated when she started the profession. Maybe no one was there for her, so she thought maybe this is the right thing, you know, you just have to learn by yourself, look after yourself.’ (Participant 7:2)

Some participants felt that they received adequate supervision and observed that this made them feel more competent than when they started their CS year. These participants expressed a sense of empowerment:

‘I am very much confident with deliveries thanks to my colleagues [*who supervised me*], which I came across when I was doing my CS. The way in which I am competent now compared with the way in which I was when I was coming from school, is totally different. Now I’m more of an independent practitioner.’ (Participant 8:4)

One participant felt that the professional nurse she worked with paved the way for her through her mentoring skills:

‘Okay so I started my comserve and I was placed at integrated management of childhood illnesses/extended programme of immunisation (IMCI/EPI), with another sister who supervised me for about plus/minus 3 weeks…during this time she definitely showed me exactly what needed to be performed with regard to filling in like the road to health booklet, she showed me exactly how to take care of the ill child …I feel like I was definitely supervised.’ (Participant 2:1)

#### Sub-theme 1.3: Participants experienced the need for more information

The participants expressed that they looked forward to their CS year because they wanted to learn more. However, they indicated that the various PHC guidelines were always changing and that what they might have learnt while in college or university could have changed once they started their CS year. Two participants expressed a desire for a short course to inform them about what was expected of a CSN and the latest developments:

‘[*B*]ecause in terms of guidelines, protocols, they do change day-in-and day-out, every day there are new developments. You can’t say things are… when you’re at school, are performed like this, when you come in the real world totally not carried out like that… when in the real world it’s practical.’ (Participant 4:6)

‘So, I do wish that there could be a certain course in all these departments to give information to CSN as to what should a professional nurse be doing there.’ (Participant 7:8)

A participant mentions the lack of resources such as information, specifically guidelines:

‘… [*R*]esources, like the availability of information, which is the guidelines, if that is available, I do not think that it would be difficult.’ (Participant 1:17)

The same participant continues:

‘I didn’t see maternity guidelines, there was only one person who had maternity guidelines. If that person was not on duty you don’t know where the guidelines are.’ (Participant 1:5)

A participant described seeking help from professional nurses:

‘So if you had to ask something you had to go and then ummm [*sic*] the person will take me ok and then we check the guidelines together.’ (Participant 1:7)

Another participant alluded to the personal connection between herself and the professional nurse from whom she sought support:

‘[*A*]s I said, as with the EPI/IMCI section there was a lot of support, I could definitely go to the sister, who is still here at the facility and I would ask her, or she would answer my concerns, she would be there for me.’ (Participant 2:3)

There was a perception that the older nurses were more knowledgeable, therefore they were approached first in terms of assistance:

‘I always interact with my colleagues, especially the older colleagues, before I do anything I consult them.’ (Participant 7:7)

Personal characteristics influence how information is transmitted and received. A participant observed that personal characteristics contributed to her experience of supervision and support from the professional nurses:

‘So, it’s not like I was having my own strange personality, where they couldn’t even tell me, no, you’re doing this wrong way, and what not. So, I think the fact that we were getting along socially, enabled me to ask them questions and it also enabled them to impart information to me.’ (Participant 8:3)

#### Sub-theme 1.4: The participants experienced a need for rotation

A participant observed that he or she had the expectation of being rotated to all departments within the facilities to which they were assigned to optimise their year of CS learning. Not all participants experienced such rotation:

‘… [*B*]ecause you’re work in the one programme for a whole year…at the clinic I was in…there is not such [*rotation happening*].’ (Participant 3:10)

Participants considered staff shortages to be the cause for the lack of rotation:

‘There was no rotation whatsoever. We were short [*of staff*] man, we understood the problem, there was no chance that we could rotate.’ (Participant 6:2)

Absenteeism of staff further impacted rotation:

‘When a certain person is not there even though you haven’t stood on your own two feet, you …are expected to go and fill that person’s space, so it was difficult.’ (Participant 1:5)

Some participants were rotated in the PHC clinics and indicated that the rotation was conducted in a systematic manner:

‘Okay, I rotated to Integrated management of childhood illnesses (IMCI) …went to the short training of IMCI …and practice what I have learnt in the training. Same in ANC (antenatal care)… I went to the basic antenatal care (BANC), and then I came back and practised, ja.’ (Participant 10:4)

‘Yes, I did [*rotate*]. When I arrived here I started at the ANC side …then was moved to the IMCI side, the children… the babies room and then allocated at the midwife obstetric unit (MOU) for about 3 months. This year, I went back to antenatal side… they did rotate me.’ (Participant 7:4)

The following participant (quoted next) indicated that rotation enabled her to become competent and instilled in her a feeling of confidence and that she was able to work anywhere in the PHC clinic:

‘So, when antenatal is not there I go and work in antenatal. When somebody…tuberculosis (TB) sister is not there, I go and work in TB because I know what is happening in TB. When somebody’s not there in EPI and IMCI, I go there and work in IMCI, because I know what is happening. I may not know everything, but I am confident I will be able to manage cases unless some of the cases are beyond my scope….’ (Participant 1:18)

In discussing the diverse needs with regard to their CS placement, seven out of the 10 participants reported not benefitting from the CS experience because of a lack of adequate supervision from professional nurses. These participants suggested that this could have been as a result of poor staffing levels in the PHC facilities as well as the high expectations of the professional nurses with regard to their capabilities as novice nurses. The participants who expressed having a positive CS experience, portrayed a work environment in which they had the support of professional nurses.

### Theme 2: Participants expressed various experiences regarding social interaction during community service at primary healthcare clinics

This study’s findings revealed that some CSNs indicated that they did not feel welcome in the facilities. In their opinion, clique formation in the clinics impacted their ability to become part of the nursing team, which appeared to lead to experiencing anxiety as they entered a new work environment, which felt ‘negative’ to them. On the other hand, participants who had positive experiences believed good relationships and interaction between the professional nursing staff and CSNs made it easier to ask questions, to which the staff reciprocated by imparting knowledge.

#### Sub-theme 2:1: Participants expressed negative experiences regarding social interaction

Participants shared their experience of not being welcomed into the clinics they were assigned to:

‘… [*T*]hey were not as welcoming in this facility…I remember overhearing one of the professional nurses saying, they send another comserve whom we must train once again; we don’t want comservers, we want people with experience … so they were not welcoming.’ (Participant 3:8)

Poor social interaction between the CSNs and the professional staff was sometimes experienced when the CSNs were seeking support:

‘People told me… even the OM [*Operational manage*] said, agh, we all had to work here alone, so I don’t know why you’re complaining. So that was kind of my experience, I was thrown in the deep end.’ (Participant 2:2)

‘Even if you have a problem you had to stand up for yourself. Even if they see you have to stand up for yourself, nobody would say, so and so is encountering a problem here and there.’ (Participant 4:7)

Two participants reported on being exposed to practice settings where clique formation clearly existed, whereby they found it difficult to fit in:

‘… In the facility where I started comserve, there were tensions there… conflicts, because some professional nurses, they were how do I put this? but there were groups, this group in conflict…ja, its cliques, ja, in conflict with another group, and there is me in between … it was not nice, I don’t know how to describe it ….’ (Participant 3:7)

‘… There’s a lot of groupings, I would say, when it comes to social interactions. And maybe that’s why it’s so difficult to find my role per se, is because of such things. You get there and people are grouped. You know …these are my friends, or the people that I like, or situations… and this is now affecting your service area….’ (Participant 5:7)

Working in an environment where clique formation existed led to the given participants self-reflecting, then acting:

‘I think, maybe as an individual, it is just a matter of trying more to be penetrable – if that is a word – but for me to be the one that maybe approaches the cliques and maybe eventually find my way: I am mostly suitable or suited in. Ja. I think that’s the best option. On the other hand, I don’t believe in cliques. (Participant 5:10)

But then I decided to change the facility… then I went to this facility, because I felt I was too stressed working there.’ (Participant 3:6)

#### Sub-theme 2:2: Participants expressed various experiences regarding positive social interaction during community service at primary healthcare clinics

There were participants who had a good experience of feeling welcome in their clinics, which they expressed in the following manner:

‘I am at ease… I know my team that I am working with, and they are very approachable. So, the first line of contact was very important for me; I was welcomed and I was very happy with that.’ (Participant 9:5)

‘… I requested to be changed to this facility in particular, because when I was a student, I was mostly allocated to this facility, so most of the sisters here, I knew them, as a student, so they were very nice to me because we had a prior relationship. So, for me it was easy adjusting, working here, because the staff knew me and they were nice to me, they tried to make my comserve to be as comfortable as possible.’ (Participant 3:7)

Those who were welcomed also experienced collegiality:

‘[*A*]nd the support that I got from them … I didn’t expect that. Because you know you hear stories where you go and I don’t have a problem with support. You can always go to any staff member … ask anything they are really willing to help you.’ (Participant 9:1)

‘So, at the beginning, being welcomed like that, and being…all the friendly smiles, all the…when we come in the morning, we all greet each other and are treated with respect.’ (Participant 9:4)

A participant explains the impact of a positive social interaction in the work environment:

‘Yes, it does help, in the sense, where you know when something is disturbing you, that has nothing to do with work – how can I put it? – when something is bothering you…you end up not concentrating in work, right. So, I feel by talking to the next person about it, then at least you get relief and then at least you know maybe what to do next about the problem so at least it gives you that relief of coming back to your work situation and performing better.’ (Participant 7:5)

The participants verbalised the importance of being part of a team by firstly being socially connected with one’s peers. What has been elicited from the study is how a negative experience of social interaction inhibits an effective learning experience for the CSN. Positive social interaction is associated with support and collaboration with peers.

## Discussion

The CSNs identified barriers to professional development while doing CS in the PHC setting. Some of the participants highlighted how a lack of adequate orientation in the clinics hindered their ability to adapt. According to Lindfors and Junttila (2014), orientation is defined as actions taken to ensure new employees are familiar with what is expected of them and the demands of the new work environment. In discussing the outcomes of effective orientation, Raju, Magabed and Chitha (2017) state that effective orientation programmes increase the confidence of the new nurse by providing the necessary support to enhance job performance. Orientation also helps the new employee to feel welcome and to develop a sense of belonging in the new environment (Lindfors & Junttila 2014; Smith-Carrington 2018).

The results of this study revealed that for the majority of the participants, lack of orientation and being left to work alone in an unfamiliar environment culminated in lost opportunities for learning and created an environment for unsafe clinical practice. Therefore, participants recommended mandatory induction and orientation programmes for all CSNs prior to being placed in their respective facilities. This view has been supported in literature. According to Rush, Adamac and Gordon (2013), a formal programme for new graduate nurses that includes a distinct orientation phase followed by experience in the setting, improves the transition experience. Chandler (2012) and Ashley, Halcomb and Brown (2016) suggest that limited orientation can be a barrier to the successful transition of a new nurse into practice.

The participants in this study experienced a lack of orientation and spoke of a lack of adequate supervision. The lack of supervision was directly related to the availability of professional nurses to supervise. Hanks (2017) states that when newly qualified nurses start their professional lives, their cognitive, psychomotor, decision-making and clinical judgement skills are formed. This is a crucial time in the professional life of a nurse; thus, adequate support is needed to facilitate the development of competent decision-making. Ten-Hoeve, Kunnen and Brouwer (2018) observe that experiencing a lack of support can lead to the novice nurse having decreased confidence and a lack of enjoyment of work. Hofier and Thomas (2016) also explain how unsupportive work environments may affect new nurses while they are trying to integrate into a new work environment, causing feelings of being overwhelmed and exhausted.

Some participants expressed anxiety over working alone as they felt prone to making clinical judgement errors because of inexperience. Hunsberger, Baumann and Crea-Arsenio (2013) explain the sense of anxiety by observing that the mentor acts as the safety net to prevent the new nurse from making errors and thus building confidence. If that safety net is absent, anxiety ensues.

Makau (2016) states that the outcome when an experienced practitioner works with an inexperienced practitioner is skills and knowledge enhancement, anxiety reduction and safe practice. Rush et al. (2013) associate the effective transition experience of the new nurse to competent practitioner with the ability to access support.

Staffing shortages inhibited the CSNs from being rotated to the different departments in the facility. Furthermore, not being able to attend training programmes prevented them from broadening their knowledge. Not only do the inadequate staffing levels affect staff development, Maillacheruvu and McDuff (2014) inform us that staffing levels directly impact the patients’ waiting time and the time spent on patient care by professional nurses.

Participants expressed a need for material resources, such as guidelines, to be more readily available. Guidelines are required to use as a reference for diagnosis and management of patients and therefore considered to be essential, particularly in the absence of a senior nurse to go to for guidance. The participants addressed the need for more knowledge to be safe practitioners. Attending courses and the availability of written guidelines could provide this knowledge. In support of this view, Coventry, Maslin-Prothero and Smith (2015) and Mlambo, Silen and Mcgrath (2021) are of the opinion that barriers to staff participation in continuing professional development should be addressed at an organisational level. Therefore, if the organisation expects safe, quality nursing care, it should invest in facilitating access to continuing professional development activities.

Aggar et al. (2017) state that not exposing the new nurse to diverse clinical experience makes them feel unchallenged and could lead to a lack of job satisfaction. Primary health care clinical rotations enhance learners’ knowledge and skills as well as demonstrate the realities of healthcare (Christensen et al. 2015).

Walker et al. (2015) observed that newly graduated nurses are aware of the steep learning curve they have commenced as new nurses. In addition, they have the expectation of adequate employer support to obtain the requisite experience and skills needed to function as a valuable member of the team. The few CSNs who benefited from being supervised spoke of a sense of empowerment because of the supervision. This view confirms the notion, as proposed by Kamphinda and Chilemba (2017) that supervision creates an environment for effective clinical learning.

Social interaction, as reported in this study, is understood to be a precursor to support. Support was indicated as being a prevalent need for CSNs in the PHC facilities in which this study was conducted. For there to be skills transfer, there must be an interdependent relationship whereby the professional nurses and CSNs collaborate. Moore, Prentice and Mquesto (2015) also found in their study that for a collaborative relationship to exist, nurses need to know each other professionally and personally. Goodare (2015) and Chipps et al. (2019) are of the opinion that the importance of being part of a team by being socially connected with one’s peers enhances the new nurses’ self-esteem.

Negative experiences were characterised as not feeling welcomed, the unfriendly way that senior colleagues spoke to them and the existence of clique formation. Mulaudzi, Libster and Phiri (2009) explain the relevance of welcoming new nurses. They state that the process of a new nurse becoming a professional nurse requires mentoring, motivating and welcoming. The authors are of the view that burnout on the part of professional nurses may be the reason for the lack of mentoring of new nurses. Some of the participants concur that the professional nurses simply did not have time because they have to manage large patient loads.

Wilson (2016) states that a sense of belonging was very important to the newly qualified nurse and that to feel they belonged and were accepted, they often adopted the behaviours and practices they experienced in the workplace.

The reported incivility experienced by two of the participants who were assigned to clinics in which clique formation existed and made it difficult for them to ‘fit in’ to the work environment, feel part of the team and led to difficulty in performing their duties because of the creation of a sense of not belonging. Woo and Newman (2019) inform on how newly graduated nurses in Singapore are impeded from fitting in their clinical settings because of clique formation, which in their experience is affiliated with ethnicity and causes newly graduated nurses not in a particular ethnic group to feel disconnected from the nursing team. Writing on the South African CSNs (newly graduated nurses) experiences of interpersonal challenges during CS, Abiodun et al. (2019) refer to a sense of loneliness and isolation experienced by CSNs who did not feel accepted in their clinical settings.

In contrast, the participants in this study who had a positive experience of social interaction all spoke of feeling part of the team and being supervised until they could work alone. The CSNs in these facilities reported having an identity in the team, of being treated like a colleague needing to be nurtured by the professional nurses.

## Limitations of the study

The study was limited to PHC facilities in NMBD. In order for transferability to occur, a thick description of the setting and research results supported by verbatim quotes from the research participants were provided in this study. This allows for similar studies to be carried out in similar settings in different districts and provinces and for results to be compared.

## Recommendations

Based on the findings of the study and reviewed literature, the following recommendations are proposed by the researchers:

- This was an exploratory qualitative study, using a small number of participants. A larger study could be performed to evaluate the experiences of CSNs in other districts and provinces in South Africa.
- There is a need for adequate staffing levels, mandatory induction programmes, guidelines and orientation programmes per facility.
- A skills audit per CSN to identify learning needs prior to commencing work. A skills checklist of the required skills proficiency needed in the PHC setting should be completed and signed by the CSN prior to a CSN commencing work in PHC.
- A policy from the National Department of health mandating that the CSNs be given the time to attend training programmes during the CS year and be rotated through all the departments within the PHC facility they have been allocated to. This would ensure adequate learning within the PHC setting.
- A senior nurse to be trained and appointed as a supervisor to work alongside the CSN in each PHC facility.
- Research regarding experiences of operational managers and professional nurses working with CSNs in the PHC setting is recommended.

## Conclusion

The study’s findings revealed that not all PHC clinics in which CSNs who participated in this study worked provided supervision and support. Based on the experiences of the CSNs, supervision and support were limited to a few PHC clinics. Recommendations based on the findings of the study have been made to assist in improving the experience of future CSNs in the PHC setting in NMBD.

## Appendix 1

**TABLE 1-A1:** Themes and subthemes.

Themes	Sub-themes
**Theme 1:** The participants experienced diverse needs related to their community service placement at primary healthcare clinics.	The participants experienced the need for: 1.1 Orientation 1.2 Adequate supervision 1.3 More information 1.4 Rotation
**Theme 2:** Participants expressed various experiences with regard to social interaction during community service at primary healthcare clinics	2.1 Participants expressed negative experiences of social interaction 2.2 Participants expressed positive experiences of social interaction
